# Supplementary material for: Role and mechanism of NCAPD3 in promoting malignant behaviors in gastric cancer
Source: Front Pharmacol. 2024 Apr 22;15:1341039. doi: 10.3389/fphar.2024.1341039 (PMC11070777; doi:10.3389/fphar.2024.1341039)
Supplement: Supplementary file 11 [file DataSheet2.ZIP › GSEA/Transcipt factor/my_analysis.Gsea.1599461814734/GR_01.html]

Details for gene set GR\_01[GSEA]

|  || Dataset | filtered\_dataset.sample\_info.cls#WT\_versus\_NCAPD3\_MUT |
| Phenotype | sample\_info.cls#WT\_versus\_NCAPD3\_MUT |
| Upregulated in class | NCAPD3\_MUT |
| GeneSet | GR\_01 |
| Enrichment Score (ES) | -0.5383582 |
| Normalized Enrichment Score (NES) | -2.2529624 |
| Nominal p-value | 0.0 |
| FDR q-value | 0.023700383 |
| FWER p-Value | 0.121 |
Table: GSEA Results Summary

  

Fig 1: Enrichment plot: GR\_01      
 Profile of the Running ES Score & Positions of GeneSet Members on the Rank Ordered List

  

| SYMBOL | TITLE | RANK IN GENE LIST | RANK METRIC SCORE | RUNNING ES | CORE ENRICHMENT || 1 | 64324 | NSD1 | 534 | 0.455 | -0.3307 | No |
| 2 | 26953 | RANBP6 | 630 | 0.406 | -0.3522 | No |
| 3 | 55003 | PAK1IP1 | 734 | 0.359 | -0.3849 | No |
| 4 | 10492 | SYNCRIP | 779 | 0.334 | -0.3781 | No |
| 5 | 667 | DST | 886 | -0.303 | -0.4194 | No |
| 6 | 80315 | CPEB4 | 1053 | -0.448 | -0.4870 | Yes |
| 7 | 11221 | DUSP10 | 1095 | -0.475 | -0.4620 | Yes |
| 8 | 604 | BCL6 | 1136 | -0.504 | -0.4328 | Yes |
| 9 | 9245 | GCNT3 | 1147 | -0.511 | -0.3814 | Yes |
| 10 | 639 | PRDM1 | 1159 | -0.523 | -0.3293 | Yes |
| 11 | 1540 | CYLD | 1162 | -0.530 | -0.2699 | Yes |
| 12 | 23657 | SLC7A11 | 1205 | -0.581 | -0.2334 | Yes |
| 13 | 10150 | MBNL2 | 1217 | -0.591 | -0.1735 | Yes |
| 14 | 4622 | MYH4 | 1355 | -0.811 | -0.1788 | Yes |
| 15 | 4734 | NEDD4 | 1369 | -0.856 | -0.0899 | Yes |
| 16 | 55729 | ATF7IP | 1392 | -1.034 | 0.0129 | Yes |
Table: GSEA details [plain text format]

  

Fig 2: GR\_01      
 Blue-Pink O' Gram in the Space of the Analyzed GeneSet

  

Fig 3: GR\_01: Random ES distribution      
 Gene set null distribution of ES for **GR\_01**
